# Improving the Diagnostic Performance by Adding Methylation Marker to Conventional Visual Examination in Identifying Oral Cancer

**DOI:** 10.3390/diagnostics12071544

**Published:** 2022-06-24

**Authors:** Cheng-Chieh Yang, Yee-Fun Su, Han-Chieh Cheng, Yi-Chen Juan, Yu-Wei Chiu, Cheng-Hsien Wu, Pei-Yin Chen, Yu-Hsien Lee, Yen-Lin Chen, Yi-Tzu Chen, Chih-Yu Peng, Ming-Yi Lu, Chuan-Hang Yu, Yu-Feng Huang, Shou-Yen Kao, Chyng-Wen Fwu, Chung-Ji Liu

**Affiliations:** 1 Department of Stomatology, Oral and Maxillofacial Surgery, Taipei Veterans General Hospital, Taipei 11217, Taiwan; ccyang1124@nycu.edu.tw (C.-C.Y.); colabear913@gmail.com (H.-C.C.); marcellinwu@gmail.com (C.-H.W.); sykao@vghtpe.gov.tw (S.-Y.K.); 2 Department of Dentistry, School of Dentistry, National Yang Ming Chiao Tung University, Taipei 112304, Taiwan; dentalhandsomeboy@gmail.com; 3 iStat Biomedical Co., Ltd., New Taipei City 22102, Taiwan; yeefun@istat.com.tw (Y.-F.S.); yichen@istat.com.tw (Y.-C.J.); 4 Department of Stomatology, Chung Shan Medical University Hospital, Taichung 40201, Taiwan; send8034@hotmail.com (P.-Y.C.); cos1018@gmail.com (Y.-H.L.); yenlin0706@gmail.com (Y.-L.C.); chenyitzu0831@gmail.com (Y.-T.C.); peng.chihyu@gmail.com (C.-Y.P.); miexyz@gmail.com (M.-Y.L.); chuanhang2007@gmail.com (C.-H.Y.); whuang@csmu.edu.tw (Y.-F.H.); 5 College of Oral Medicine, Chung Shan Medical University, Taichung 40201, Taiwan; 6 Department of Oral and Maxillofacial Surgery, MacKay Memorial Hospital, Taipei 10449, Taiwan

**Keywords:** DNA methylation, oral cancer, oral epithelial dysplasia, visual oral examination, *ZNF582*

## Abstract

Background: Visual oral examination (VOE) is a conventional oral cancer screening method. This study aimed to evaluate the value of methylation marker to assist VOE in identifying oral epithelial dysplasia and oral squamous cell carcinoma (OED/OSCC) from non-cancerous lesions in a real-world situation. Methods: 201 patients with high-risk personal habits who self-perceived oral anomaly were VOE examined, *ZNF582* methylation (*ZNF582^m^)* tested, and histologically diagnosed. Results: Among them, 132 patients (65.7%) were histologically diagnosed OED/OSCC. Using VOE, 56.1% OED/OSCC patients had possible oral cancer, whereas 37.7% non-OED/OSCC patients had leukoplakia. *ZNF582^m^*-positive was detected in 90.2% OED/OSCC patients and 44.9% non-OED/OSCC patients. Various logistic regression models were postulated to evaluate the diagnostic performance of conventional VOE and new strategies using *ZNF582^m^*. ROC analysis and its corresponding C-index demonstrated that either triage or co-testing models of VOE and *ZNF582^m^* could improve diagnostic performance and discriminative abilities compared with the VOE only approach. Conclusions: In conclusion, methylation marker test shows equivalent performance to an experienced judgment by oral maxillofacial surgeons and plays a significantly supplementary role in increasing the efficacy in identifying oral malignant lesions. *ZNF582^m^* may be an especially important tool for family physicians or general dentists to properly diagnose suspicious oral lesions.

## 1. Introduction

Oral squamous cell carcinoma (OSCC) is one of the most common head and neck cancers and accounts for more than 90% of oral malignant diseases. OSCC is preceded by oral lesions, termed oral potentially malignant disorders (OPMDs), according to the WHO classification [1]. OPMDs are heterogeneous lesions, including leukoplakia, erythroleukoplakia, proliferative verrucous leukoplakia, erythroplakia, and verrucous hyperplasia, associated with an increased risk of malignant transformation to cancer. These oral potentially malignant lesions could be asymptomatic and may be diagnosed as benign lesions through their clinical appearance.

Early detection of OPMDs with high malignant transformation possibility is crucial for clinicians to treat and monitor oral cancers. At present, visual oral examinations (VOE) are the routine screening method exerted subjectively by clinicians in identifying oral cancerous lesions. However, VOE alone posed a high positivity rate, due to the shortcomings such as detecting obscure lesions, inflammatory ulcers, or immune-related lesions and distinguishing between benign, potentially malignant disorders, or real cancerous lesions even though VOE was performed by professional specialists [2,3,4,5,6,7]. A global oral cancer forum (GOCF) in 2016 suggested that the interpretation of VOE results may greatly depend on the competence of the examiner and requires training and calibration of screeners. Therefore, depending solely on VOE by clinicians to distinguish various OPMDs with diverse clinical features may increase the risk of missing early malignant lesions [2,3,8]. An analysis by the Taiwan Health Promotion Administration demonstrated that the VOE positivity rate differs across healthcare units, including medical centers, regional hospitals, district hospitals, and local clinics. Medical centers had the highest VOE-detected positive rates, likely because of the deep-rooted stereotypes that patients would receive better medical attention in large medical centers. Patients aware of or being detected with suspected OPMD or cancerous lesions are usually referred to medical centers for further management. Regional and district hospitals had lower VOE positivity rates and were comparable to each other. In contrast, the local clinics produced the lowest VOE positivity rates possibly due to the lack of experience and resources in performing oral lesion screening [9]. Thus, there is a need of reliable molecular markers that can be auxiliary to the conventional VOE method to effectively capture the cancerous lesion at an early stage [2,3,8]. A well-characterized standard testing regimen such as *ZNF582* methylation (*ZNF582^m^*) may play an important role in assisting the general healthcare providers in identifying patients with a high suspicion of cancerous lesions.

Oral biopsy examination remains the current gold standard for diagnosing oral cancer when a suspicious OPMD is detected. OPMDs with oral epithelial dysplasia (OED) often progressed into carcinomas more frequently than OPMDs without dysplasia [10,11,12]. Nevertheless, many patients fear and develop anxiety about taking biopsies repeatedly for diffuse lesions or later-occurred lesions during the follow-up period [13,14]. The study of P.J. Thomson investigated 26 patients presenting with unilateral OSCC or premalignant lesions during VOE procedures, and biopsies were taken from the clinically normal-looking mucosa of their corresponding contralateral sites. Fifteen out of twenty-six patients (58%) had histologically abnormal tissue, including benign cellular atypia, dysplasia, and SCC. The study implies that VOE is limited by its subjectively observational nature and urged the requirement of more reliable and objective testing regimen for the epithelial behaviors manifesting the processes of cancer development [15]. The 2016 GOCF also commented that although many studies investigated the usefulness of oral cancer screening tests, there were relatively few reviews of screening programs and only one community-based randomized control clinical trial implemented in India from 1996 to 2004 [3,16,17,18,19,20]. In addition, according to the U.S. CDC report on oral cancer incidence in the U.S. from 2007 to 2016, new oral cancer cases did not seem to have reduced but instead consistently increased over the years [21]. The evidence demonstrates that the current oral cancer screening using VOE may not be able to capture all high-risk lesions at their early stage and thus fails to reduce oral cancer incidence and mortality [3].

DNA methylation is one of the best-studied epigenetic regulations that play a crucial role in cancer development. It involves chemical modification of cytosine (C) to 5-methylcytosine (5mC) in GC-rich regions on promoters of regulatory regions of many genes [22]. Cumulative studies have shown that aberrant DNA methylation represses key tumor suppressor genes or regulatory regions within the genome, leading to cell growth dysregulation or altered responses to cancer therapies [23,24]. Several methylated genes have been demonstrated as clinically effective biomarkers for detecting cancers such as bladder, colon, cervical, and oral cancer [25,26,27,28,29]. As a result, they have received regulatory market approval as DNA methylation-based in vitro diagnostics (IVDs). *ZNF582^m^*, with the product name of ZNF582 DNA Detection Kit (iStat Biomedical, New Taipei City, Taiwan), is a Conformité Européene in vitro diagnostic (CE-IVD), which has been used as an adjunctive tool assisting VOE for oral cancer by clinicians to facilitate decision making on whether a histopathological examination should be performed immediately [8,25,29,30]. Several hypermethylated genes have been determined in human oral squamous cell carcinoma (OSCC) [31]. Among those hypermethylated genes, *ZNF582* was studied rigorously and was selected for biomarker development based on promising findings. The correlation between *ZNF582* methylation and oral cancer has been extensively studied in recent years [29,30,32,33,34,35]. *ZNF582* methylation demonstrated as an effective biomarker for the detection of oral dysplasia and oral cancer via collection of oral scrapings from normal oral mucosa subjects, oral potentially malignant patients, and OSCC patients. Methylation of *ZNF582* may be applicable as a triage tool for patients with abnormal visual oral examinations [29]. Furthermore, a recent follow-up study has also shown that high incidence of OSCC and oral disease progression was observed in patients with high *ZNF582* methylation lesions at baseline [34].

Given the limitation of VOE, we evaluated the added value of *ZNF582* methylation for VOE in identifying OED/OSCC from a diverse state of OPMDs.

## 2. Materials and Methods

### 2.1. Study Design and Study Population

A multicenter study was conducted at three teaching hospitals, including Taipei Veterans General Hospital (TVGH), Mackay Memorial Hospital (MMH), and Chung Shan Medical University Hospital (CSMUH), from 2019 to 2020 in Taiwan. The Institutional Review Boards at each participating hospital (IRB No: TVGH(2019-09-002A), MMH(19CT007be), and CSMUH(CS18221)) reviewed and approved this study. The experiments were undertaken with the understanding and written consent of each subject and that the study conforms with The Code of Ethics of the World Medical Association (Declaration of Helsinki) printed in the British Medical Journal (2013).

Eligible patients were 20 years or older with collectible epithelial cells in the oral cavity. They sought medical care at the participating hospital; had a high-risk personal habits of cigarette smoking, alcohol drinking, or betel nut chewing (included quitter); or had self-perceived oral mucosa abnormalities. Patients were enrolled if they fulfilled the inclusion criteria and signed the written informed consent. Pregnant patients and those with previous malignancy were excluded. All patients included in this study had histologically confirmed oral lesions and did not have a recent systemic inflammatory state, immune disease, or any acute infection.

### 2.2. Clinical Evaluations

Demographic data, family history, personal habits, and reasons for seeking health cares were obtained by well-trained study nurses. Patients were then examined via conventional VOE by experienced senior oral maxillofacial surgeons (OMS). The VOE assessment of leukoplakia, erythroleukoplakia, erythroplakia, verrucous hyperplasia, and possibly oral cancer were identified as abnormal findings in this analysis.

After physical examinations, the oral exfoliated cell specimen from the mucosal lesion was collected by oral swab of each patient. In addition, a biopsy specimen was taken from suspected lesions. A pathologist at each respective hospital reviewed the biopsy specimens and made a diagnosis through histopathological analysis. The diseases of interest in this analysis, i.e., oral epithelial dysplasia and oral squamous cell carcinoma (OED/OSCC), included the histopathological diagnoses of mild dysplasia, moderate dysplasia, severe dysplasia, carcinoma in situ, and OSCC.

### 2.3. DNA Preparation of the Oral Specimen

Oral epithelial cells (OEC) were collected using a foam brush and preserved in “EpiGene” Specimen Collection and Transfer Tube (iStat Biomedical Co., Ltd., New Taipei City, Taiwan; MOHW-MD-(I)-No. 006315). Subsequently, genomic DNA (gDNA) was extracted from the OECs with the “Epigene” Nucleic Acid Extraction kit (iStat Biomedical Co. Ltd., New Taipei City, Taiwan; CE-IVD), according to the manufacturer’s written instructions.

### 2.4. ZNF582 Methylation (ZNF582^m^) Assay

The concentration of extracted gDNA was quantified using the NanoDrop 2000c Spectrophotometer (Thermo Fisher Scientific, Wilmington, DE, USA) and was subsequently subjected to bisulfite conversion using the “Epigene” Bisulfite Conversion Kit (iStat Biomedical Co., Ltd., New Taipei City, Taiwan; MOHW-MD-(I)-No. 006611 and CE-IVD). This process converts the unmethylated cytosine of the gDNA to uracil, while the methylated cytosine remains unchanged.

The bisulfite-converted gDNA was then used for *ZNF582^m^* assays. Briefly, *ZNF582^m^* levels were determined using the “Epigene” ZNF582 DNA Detection Kit (iStat Biomedical Co., Ltd., New Taipei City, Taiwan; CE-IVD) through quantitative methylation-specific PCR by using TaqMan-based technology with Light Cycler LC480 (Roche Applied Science, Penzberg, Germany). The type II collagen 2A gene (*COL2A*), which contains non-CpG sequences, was used as the internal reference and the validity indicator. Two crossing point (Cp) values were determined, namely one from *ZNF582* and the other from *COL2A*, and the DNA methylation was qualitatively determined in accordance with manufacturer’s instructions.

### 2.5. Statistical Analysis

The continuous and dichotomous variables are expressed as mean with standard deviation (SD) and number with percentage, respectively. The chi-square test for categorical variables and the Mann–Whitney U test for continuous variables were used to examine the differences in distributions of patient characteristics between patients with and without confirmed OEC/OSCC.

Various logistic regression models were first specified to predict the probability of OEC/OSCC and to evaluate the diagnostic performance of VOE and the *ZNF582^m^* marker. The “reference model” was adjusted for age, sex, and betel nut chewing, which were important and established risk factors for OSCC. Subsequent models were created by adding one or two additional covariate(s) to the reference model. The “VOE model” and “MET model” were based on the reference model and added the additional VOE and methylation marker covariate, respectively. The “triage (sequential) model” was developed by adding an indicator, identifying patients with abnormal VOE findings and methylation positivity, to the reference model. The “co-testing (parallel) model” was constructed by adding both VOE and methylation marker to the reference model.

We then generated the receiver operating characteristic (ROC) curves and estimated the area under the curve (AUC; also known as C-index) with a 95% confidence to assess the discriminative ability for each model. The values of the C-index could be classified as poor (<0.7), acceptable (≥0.7 and <0.8), and excellent (≥0.8) discrimination [36]. In addition, the C-index difference, integrated discrimination index (IDI), and relative IDI were further used to describe the improved predictive performance between models. IDI is a widely used statistical method to estimate whether an investigated diagnostic marker provides additional diagnostic value beyond traditional clinical information [37].

Statistical analyses were conducted using Statistical Analysis System software (SAS System for Windows, Version. 9.4; SAS Institute, Cary, NC, USA). The level of statistical significance was set at *p* < 0.05.

## 3. Results

Among 201 patients, 65.7% (132 patients) were diagnosed with OED/OSCC, and 34.3% (69 patients) were non-OED/OSCC. Overall, there were 82.6% males; 19.6% had a college degree or higher; 8.5% had a family history of oral cancer; and 79.6%, 74.5%, and 51.7% were cigarette, betel nut, and alcohol ever and/or current users, respectively. Furthermore, 43.3% had all three habits, and 32.8% had any two, while 9.0% had only one of these habits, and 14.9% were never exposed to cigarette, betel nut, and alcohol. The mean age was 56.3 ±12.0 years.

Regarding clinical manifestations, 51.7% had abnormal swelling, thickening, or unusual pigmented plaques, while 45.8% experienced pain, non-healing ulcers, the presence of erosions, and lumps in the oral mucosa. Further, 84.2% had abnormal findings by VOE, including 23.4% leukoplakia, 19.5% erythroleukoplakia/erythroplakia/verrucous hyperplasia, and 41.3% with possible oral cancer, respectively. In addition, 74.6% had positive methylation results. No significant difference of the VOE judgment among the participated OMS from the three study sites.

The comparisons of patient characteristics by OED/OSCC status are shown in Table 1. Patients with OED/OSCC were older (*p* = 0.001), more likely male (*p* = 0.01), had a lower education level (*p* < 0.001), were more likely to have betel-nut-chewing behavior (*p* = 0.04), and more likely experienced pain, non-healing ulcers, presence of erosions, and lumps in the mucosa (*p* < 0.001).

The distribution of VOE results between the two groups, i.e., the histopathological verification as OED/OSCC or not, was also significantly different. In the OED/OSCC group, there were 21 (15.9%) leukoplakia, 14 (10.6%) erythroplakia/erythroleukoplakia, 12 (9.1%) verrucous hyperplasia, 74 (56.1%) possible oral cancer, 8 (6.1%) ulcer, and 3 (2.3%) others using visual examination. On the other hand, in the non-OED/OSCC group, there were 26 (37.7) leukoplakia, 6 (8.7%) erythroplakia/erythroleukoplakia, 7 (10.1%) verrucous hyperplasia, 9 (13.0%) possible oral cancer, 7 (10.1%) ulcer, and 14 (20.3%) others. Moreover, nearly 90% of patients with OED/OSCC were tested positive for the methylation marker *ZNF582^m^*, whereas only 31 patients without OED/OSCC at the biopsied lesion sites had positive methylation results. However, they are more likely to show hyperplasia and hyperkeratosis in the histopathological examination and clinical features of swelling, mucosal thickening, or pigmentation.

**Figure 1:**
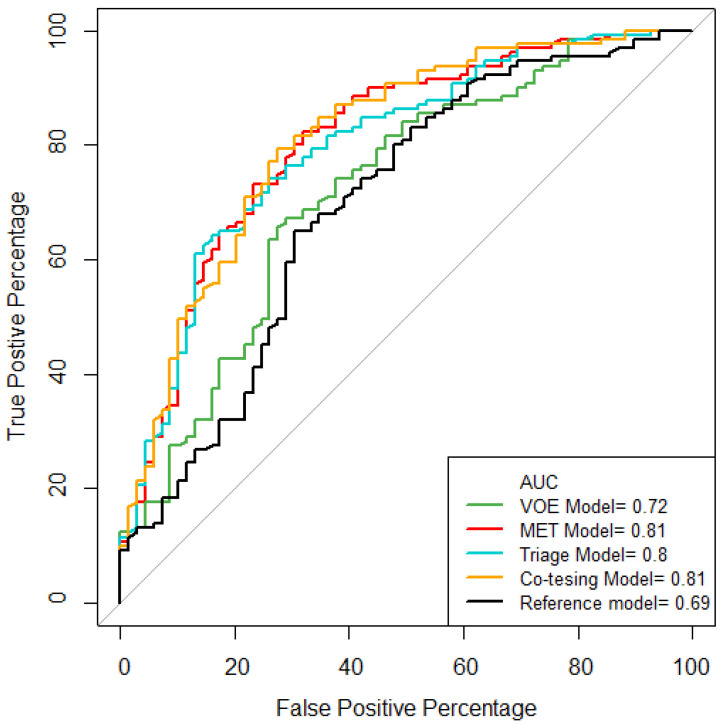
ROC curves from various models.

The C-index of the reference model was not quite satisfactory (0.69; 95% CI; 0.61–0.77; Figure 1). However, as seen in Table 2, the excellent predictive performances to discriminate OED/OSCC were observed in the MET model (C-index, 0.81; 95% CI, 0.74–0.87), triage model (0.80; 0.73–0.86), and co-testing model (0.81; 0.75–0.88). While comparing to the reference model, the MET model (*p* = 0.001), triage model (*p* = 0.004), and co-testing model (*p* = 0.002) had significantly better C-indices but not the VOE model (*p* = 0.26). Additionally, significantly higher discriminative abilities were observed for the triage model (*p* = 0.01) and the co-testing model (*p* = 0.004) compared to the VOE model.

Variables adjusted in the VOE model include age, sex, betel nut chewing, and visual oral examination results. Variables adjusted in the MET model include age, sex, betel nut chewing, and methylation marker. Variables adjusted in the triage model include age, sex, betel nut chewing, and an indicator to identify patients with positive results based on visual oral examination and methylation positivity. Variables adjusted in the co-testing model include age, sex, betel nut chewing, visual oral examination results, and methylation marker. Variables adjusted in the reference model include age, sex, and betel nut chewing.

Concerning improving predictive performance in diagnosing OED/OSCC based on IDI, results were consistent with findings from the C-index (Table 3). Compared to the reference model, the co-testing model had the greatest improvement (IDI, 18%; 95% CI, 12–24%; *p* < 0.001), followed by the MET model (17%; 11–22%; *p* < 0.001) and triage model (14%; 8–18%; *p* < 0.001). Consistently, the co-testing model (IDI, 14%; 95% CI, 19–20%; *p* < 0.001) and triage model (10%; 5–14%; *p* < 0.001) were also significantly better than the VOE model in terms of IDI. A similar trend was observed between models while using relative IDI to indicate the improvement of diagnostic performances for predicting OED/OSCC.

**Figure 2:**
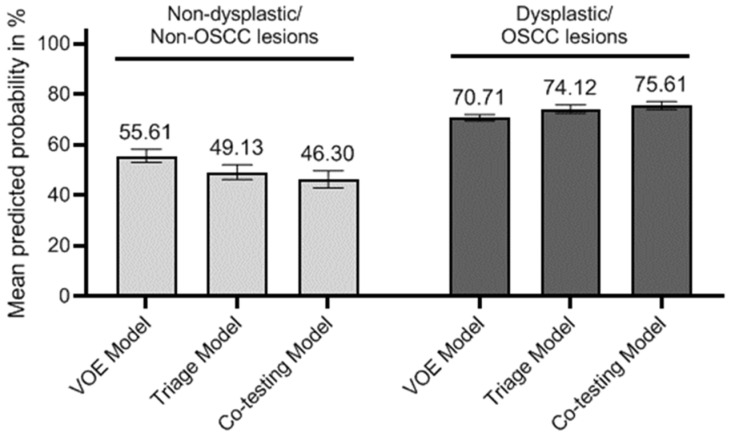
Comparisons of mean predicted probability among models by OED/OSCC status.

Figure 2 showed comparisons of mean predicted probability among various models. For example, in dysplasia or OSCC lesions, the mean predicted probability increased from 70.7% in the VOE model and 74.1% in the triage model to 75.6% in the co-testing model. Conversely, in non-OED/non-OSCC lesions, the probability also “improved” from 55.6% in the VOE model and 49.1% in the triage model to 46.3% in the co-testing model since, clinically, we expected a better model should have a lower predicted probability in this group. Interestingly, compared to the VOE model, the improvement for non-OED/non-OSCC lesions was twice the number for OED/OSCC lesions in triage (12% vs. 5%) and the co-testing (17% vs. 7%) models, respectively.

## 4. Discussion

This study evaluated the added value of *ZNF582^m^* for VOE in identifying OED/OSCC from a diverse state of OPMDs in real-world clinical practices. Logistic regression models with adjustment of age, sex, and betel nut chewing were used to develop the diagnostic prediction model using the conventional method (i.e., VOE model) and new strategies (MET, triage, and co-testing models). ROC analysis and its corresponding C-index demonstrated that our proposed new strategies could improve diagnostic performance and discriminative abilities compared to the existing traditional VOE approach. IDI measures suggested approximately 10.0% improvement in diagnostic accuracy in triage and 14.2% in co-testing strategies. To our knowledge, this is the first study that proposed adding a well-characterized molecular marker to the traditional VOE approach in order to increase the predictive power in identifying the OED/OSCC lesions in a real-world setting.

OPMD is a heterogeneous group of oral mucosal lesions associated with an increased risk of malignant transformation to cancer. OPMDs have a complex clinical manifestation that is often ambiguous and difficult to distinguish from reactive, inflammatory, or various immune-related disease conditions of the oral mucosa [38,39]. Moreover, they could be asymptomatic in the early stage of malignant transformation and usually be assumed as benign lesions by their appearance recognized by patients and clinicians, resulting in diagnostic delay [40]. To test the feasibility and efficiency of the combination use of *ZNF582^m^* and VOE to identify OED/OSCC cases, we included a broad spectrum of OPMD and suspicious lesions in this study. The diversity of patient clinical characteristics differed from other studies, where many of those recruited OSCC or dysplastic patients were derived from specific OPMD lesions as cases and healthy participants without visible oral lesions as controls [41]. Yet, based on the multistep oral carcinogenesis process, complex oral manifestations are anticipated in the real-world situations. The diverse complaints of this study population included 51.7% (N = 104) of abnormal swelling, thickening, or unusual pigmented plaques, and 45.8% (N = 92) experienced pain, non-healing ulcers, presence of erosions, and lumps in the oral mucosa. Intriguingly, we observed that 34 out of 104 (33%) patients with abnormal swelling, thickening, or unusual pigmented plaques were non-OED/OSCC lesions. This result suggests that nearly one-third of abnormal oral manifestation is not associated with OPMD or cancerous lesions when the patients were referred or recruited in medical centers. It further demonstrates the difficulties of discriminating oral lesions merely by oral clinical manifestation that results in judgment discrepancy. In addition to the 84.2% of OPMDs, composed of 23.4% of leukoplakia, 19.5% of erythroleukoplakia/erythroplakia/verrucous hyperplasia, and 41.3% possible oral cancer, this study also recruited patients with multiple types of indistinguishable oral clinical features or symptoms that demonstrate objective and representative of the real patient population who would visit medical centers for their oral mucosal problems.

Cumulative studies have demonstrated the association between cancer and DNA methylation [42]. All 201 patients were biopsy-confirmed and categorized into OED/OSCC (N = 132) and non-OED/OSCC (N = 69) groups. Consistent to the previous studies that concluded that *ZNF582^m^* positivity was highly correlated with OED/OSCC, 119 out of 132 (90.2%) of OED/OSCC patients were *ZNF582^m^*-positive. However, 31 out of 69 (44.9%) of non-OED/OSCC were *ZNF582^m^*-positive as well. These could be explained by the field cancerization effects imposed by various carcinogens exposures, as the genetic or epigenetic alterations associated with carcinogenesis may have already existed and were detectable but not yet been observed phenotypically by the histopathologic examination [43]. For this group of patients, a prospective follow-up study is ongoing to evaluate the malignant transformation rate and the prognosis of the lesions. Our previous studies have demonstrated that *ZNF582* hypermethylation at the adjacent normal oral mucosa in OSCC patients was associated with aggressive progression and poor prognosis [29,30]. Furthermore, a high *ZNF582^m^* level was independently associated with a higher risk of malignant transformation [34]. Another reason for this observation may originate from the quality and quantity of the sampled specimen, which influenced the results interpreted by the pathologists [44]. Intriguingly, 13 out of 132 (9.9%) of OED/OSCC patients were *ZNF582^m^*-negative. This was possibly due to the insufficient collection of oral exfoliated epithelial cells in areas of the oral cavity that are difficult to reach during specimen collection or the tumor heterogeneity in a specific patient population whose epithelial cells were derived from a non-*ZNF582*-hypermethylated molecular mechanisms. This merits further investigation.

Oral examination consisting of visual inspection and palpation has been the conventional method of screening for OPMDs and oral cancer. Visual inspection surveils the whole oral cavity for any ulcerations, nodules, swelling, or alterations in color and textures. Palpation is a form of tactile examination on facial bones and soft tissues to note asymmetries or masses and the relevance of lymphadenopathy [45,46]. VOE, however, has drawbacks and depends heavily on the experience of the clinicians because some OPMDs, such as white or red lesions or persistent ulcers, are often indistinguishable with other similar lesions at their clinical presentation [2]. Therefore, a subjective interpretation occurs and may result in inappropriate medical judgment. Therefore, it is not surprising that the VOE model had a lower AUC of 0.72 than 0.80 and 0.81 in the triage and co-testing models. Namely, introducing an objective measure of the *ZNF582^m^* increased the discrimination ability for OPMD by 8% in the triage model and 9% in the co-testing model, respectively. This result implies that using DNA methylation level, such as *ZNF582^m^*, benefits the discrimination and clarification of the disease severity in terms of the malignancy from a complex setting of oral lesions. However, the sequential “triage” algorithm and the parallel “co-testing” method are different in their medical costs, especially since the sequential procedure applies the *ZNF582^m^* test in VOE-abnormal patients only. In contrast, both VOE and *ZNF582^m^* tests should be used in all patients with mucosal lesions for a parallel approach.

In addition to AUC, we used the IDI index to determine the improvement after including the *ZNF582^m^* test against the VOE only model in OED/OSCC patients and non-OED/OSCC patients separately. Our data show the inclusion of the *ZNF582^m^* test could prevent 6.5% and 9.3% of non-OED/OSCC patients from misdiagnosis and further unnecessary aggressive medical intervention in both triage and co-testing approaches, accordingly. In OED/OSCC patients, a 3.4% improvement in triage and 4.9% in co-testing strategy were also observed. This result suggested that the value-added by *ZNF582^m^* is even more powerful and significant in ruling out a non-diseased status.

In this study, all patients were examined by experienced OMS in medical centers, a group of highly trained professional surgeons to identify and treat oral mucosa and OED/OSCC lesions. However, it is still inevitable that some lesions may go unnoticed or are misdiagnosed as benign or more severe diseases. The situation is expected to be more serious in regional hospitals and community-based clinics, where the expertise in OED/OSCC detection is usually deficient. In this study, we have shown the objective *ZNF582^m^* test could play a significantly supplementary role in increasing the efficacy in identifying OED/OSCC lesions compared to the VOE-only approach. It implies the *ZNF582^m^* test can make a greater contribution to general practices of VOE in community-based health services. In Taiwan, the government provides free oral mucosal examination biennially to Taiwan nationals with high-risk personal habits in order to capture early OED/OSCC lesions. However, VOE is the only screening method, and individuals with suspected positive results are referred to an OMS or otolaryngologist for confirmatory biopsy-based diagnosis and treatment [47]. The inefficient screening procedures and the cumbersome and lengthy referral processes may delay the treatment. In the study by Chiang et al., a substantial difference in the levels of training and experience between general dentists, physician, and experienced surgeons in identifying and treating OPMD and cancerous lesions was found [48]. An Australian study also found that lack of confidence due to insufficient training was a barrier to general dentists in making accurate diagnoses [49], suggesting that additional diagnosis tools are critically needed to improve diagnosis. Thus, the well-characterized *ZNF582^m^* may be an excellent approach to help the community-based healthcare providers to clarify obscure oral lesions and make proper medical decisions regarding whether to refer a patient with suspicious lesions to an experienced OMS or otolaryngologist for further management.

This study also bears limitations. The evidence from a relatively small sample size of 201 in three hospitals may not be strong enough to show the prediction ability of these models. Thus, it warrants a further larger nationwide or even multinational studies to validate its performance. On the other hand, betel nut chewing is the major risk factor of oral cancer, followed by cigarette smoking and alcoholic drinking in Taiwan. Betel nut chewing is also endemic in South Asia and Southeast Asia countries [50]. Therefore, our strategy of incorporating *ZNF582^m^* may apply to other Asian countries with similar risk factors but not in Western countries [50]. Firstly, betel nut chewing is not popular in Western countries; secondly, the study did not evaluate other oral-cancer-causing factors such as HPV infection, which is one of the major risk factors of oral cancer in Western countries [51]. The significance of *ZNF582^m^* in HPV-related oral cancer remains to be determined. Furthermore, the inherited epigenetic background may also differ between Asian and Western or other ethnicities [52]. Therefore, whether the models incorporating *ZNF582^m^* can apply in Western countries remains unknown and requires further investigation. Another unavoidable limitation in such studies is that the clinical setting is usually more complicated than mathematic models even after considering many patient characteristics.

However, our prediction models incorporating *ZNF582^m^* has its clinical application in many perspectives. First, the triage (sequential) model suggests methylation marker could play a significant adjunctive role in the identification of OED/OSCC and may be beneficial to the current oral screening program in Taiwan. The incorporation of *ZNF582^m^* triage model into the current screening program can be applied when a positive VOE patient is identified during an oral cancer screening even when an ambiguous OPMD is observed. The *ZNF582^m^* assists the front-line clinicians to decide whether a referral or an invasive biopsy examination is required. Thus, our triage model can greatly reduce the burden and misdiagnosis risks of the front-line healthcare providers. Second, it can be useful in many rural areas with shortage of dental and medical service resources regardless whether it is in rural areas in Taiwan or other low- and middle-income countries in South Asia or Southeast Asia [53,54]. Particularly, our findings are more likely to be appreciated by OMS and otolaryngologists, general dentists, and even other medical or healthcare professionals. Considering the simplicity of sample collection, *ZNF582^m^* is measured from the oral exfoliated cells that are conveniently collected through mucosa swabbing. The harvested cell specimens are easily stored in a preservation buffer and transported to a centralized clinical laboratory for a standardized test. Test results can be obtained within few days; its data-oriented analysis is more objective and scientifically sound than that of naked-eye observation. Thus, it is beneficial for the different degrees of experience and training background of the healthcare professionals.

## 5. Conclusions

This study has developed and analyzed several prediction models based on the adjustment of several patient characteristics to assess the value of *ZNF582^m^* added to VOE in identifying OED/OSCC from a diverse state of OPMDs in real-world clinical practices. The objective nature of *ZNF582^m^* test significantly increased the efficacy to identify OED/OSCC lesions compared to the VOE-only approach. In conclusion, the *ZNF582^m^* biomarker is as good as an experienced judgment by a professional OMS and would greatly improve the efficiency in identifying malignant oral lesions. In this aspect, *ZNF582^m^* may be an especially important tool for family physicians or general dentists to properly diagnose suspicious oral lesions.

## Figures and Tables

**Table 1:** The distribution of patient characteristics by OED/OSCC status.

Variables	Overall (N = 201)	OED/OSCC		*p*-Value
		Yes (N = 132)	No (N = 69)	
Age, mean (SD), years	56.3 (12.0)	58.3 (11.7)	52.5 (12.3)	0.001
Age group, n (%)				0.03
<40	14 (7.0)	5 (3.8)	9 (13.0)	
40–49	56 (27.9)	32 (24.2)	24 (34.8)	
50–59	54 (26.9)	39 (29.6)	15 (21.7)	
60–69	47 (23.4)	33 (25.0)	14 (20.3)	
≥70	30 (14.9)	23 (17.4)	7 (10.1)	
Sex group, n (%)				0.01
Male	166 (82.6)	116 (87.9)	50 (72.5)	
Female	35 (17.4)	16 (12.1)	19 (27.5)	
Education, n (%)				<0.001
Middle school or less	80 (40.2)	63 (48.1)	17 (25.0)	
High school	80 (40.2)	51 (38.9)	29 (42.7)	
College and more	39 (19.6)	17 (13.0)	22 (32.4)	
Family history of oral cancer, n (%)				0.32
Yes	17 (8.5)	13 (9.9)	4 (5.8)	
No	183 (91.5)	118 (90.1)	65 (94.2)	
Cigarette smoking, n (%)				0.07
Nonuser	41 (20.4)	23 (17.4)	18 (26.1)	
Current user	117 (58.2)	75 (56.8)	42 (60.9)	
Former user	43 (21.4)	34 (25.8)	9 (13.0)	
Betel nut chewing, n (%)				0.04
Nonuser	51 (25.5)	26 (19.9)	25 (36.2)	
Current user	45 (22.5)	32 (24.4)	13 (18.8)	
Former user	104 (52.0)	73 (55.7)	31 (44.9)	
Alcohol drinking, n (%)				0.20
Nonuser	97 (48.3)	58 (43.9)	39 (56.5)	
Current user	75 (37.3)	52 (39.4)	23 (33.3)	
Former user	29 (14.4)	22 (16.7)	7 (10.1)	
Chief complaint I, n (%)				
Abnormal swelling, thickening, or unusual pigmented plaques	104 (51.7)	70 (53.0)	34 (49.3)	0.61
Chief complaint II, n (%)				
Pain, non-healing ulcers, presence of erosions and lumps in the mucosa	92 (45.8)	72 (54.6)	20 (29.0)	<0.001
Months of symptom median (Q1, Q3), [min, max]	2.0 (1.0, 12.175) [0.033, 121.75]	2.0 (1.0, 6.0) [0.033, 121.75]	3.0 (0.7, 12.2) [0.067, 121.75]	0.33
Visual oral examination, n (%)				<0.001
Leukoplakia	47 (23.4)	21 (15.9)	26 (37.7)	
Homogeneous thin leukoplakia	18	6	12	
Homogeneous thick leukoplakia	24	11	13	
Non-homogeneous leukoplakia	5	4	1	
Erythroleukoplakia/Erythroplakia	20 (10.0)	14 (10.6)	6 (8.7)	
Verrucous hyperplasia	19 (9.5)	12 (9.1)	7 (10.1)	
Possible oral cancer	83 (41.3)	74 (56.1)	9 (13.0)	
Ulcer	15 (7.5)	8 (6.1)	7 (10.1)	
Others	17 (8.5)	3 (2.3)	14 (20.3)	
Oral submucous fibrosis	2	0	2	
Lichen planus/Inflammation	6	1	5	
Unspecified *	9	2	7	
*ZNF582* methylation test, n (%)				<0.001
Positive	150 (74.6)	119 (90.2)	31 (44.9)	
Negative	51 (25.4)	13 (9.9)	38 (55.1)	
Biopsy results, n (%)				
Inflammation	6 (3.0)		6 (8.7)	
Atypical epithelial cell	2 (1.0)		2 (2.9)	
Hyperplasia	12 (6.0)		12 (7.4)	
Hyperkeratosis	33 (16.4)		33 (47.8)	
Mild dysplasia	21 (10.5)	21 (15.9)		
Moderate dysplasia	16 (8.0)	16 (12.1)		
Severe dysplasia	5 (2.5)	5 (3.8)		
OSCC/Carcinoma in situ	90 (44.8)	90 (68.2)	
Others **	16 (8.0)		16 (23.2)	

*p*-Value are calculated by chi-square or Mann–Whitney U test as appropriate; * unspecified: lumps or unclear or unspecified lesions identified by visual oral examination (VOE); ** including muscular-adipose tissue, fibroma, fibroepithelial polyp, pyogenic granuloma, giant cell fibroma, papilloma, fibrosis, diffuse large B-cell lymphoma, and mucoepidermoid carcinoma; OED, oral epithelial dysplasia; OSCC, oral squamous cell carcinoma.

**Table 2:** Comparisons of diagnostic performances of four models in predicting OED/OSCC.

Model ^a^	VOE Model		MET Model		Triage Model		Co-Testing Model	
	Estimate (95% CI)	*p*-Value	Estimate (95% CI)	*p*-Value	Estimate (95% CI)	*p*-Value	Estimate (95% CI)	*p*-Value
C-index	0.72 (0.64, 0.80)	<0.001	0.81 (0.74, 0.87)	<0.001	0.80 (0.73, 0.86)	<0.001	0.81 (0.75, 0.88)	<0.001
C-index difference ^b^	0.03 (−0.02, 0.07)	0.26	0.11 (0.04, 0.18)	0.001	0.10 (0.03, 0.17)	0.004	0.11 (0.04, 0.20)	0.002
C-index difference ^c^					0.08 (0.02, 0.13)	0.01	0.09 (0.03, 0.15)	0.004

^a^ Variables adjusted in reference model include age, sex, and betel nut chewing; variables adjusted in VOE model include age, sex, betel nut chewing, and visual, oral examination results; variables adjusted in MET model include age, sex, betel nut chewing, and methylation marker; variables adjusted in triage model include age, sex, betel nut chewing, and an indicator to identify patients with positive results based on both visual and oral examination and methylation positivity; variables adjusted in co-testing model include age, sex, betel nut chewing, visual, oral examination results, and methylation marker; ^b^ compared to reference model; ^c^ compared to VOE model; OED, oral epithelial dysplasia; OSCC, oral squamous cell carcinoma.

**Table 3:** Improvement in diagnostic performances of four models in predicting OED/OSCC.

Model ^a^	VOE Model		MET Model		Triage Model		Co-Testing Model	
	Difference of Mean Predicted Probability	% of Improvement	Difference of Mean Predicted Probability	% of Improvement	Difference of Mean Predicted Probability	% of Improvement	Difference of Mean Predicted Probability	% of Improvement
Compared with reference model								
IDI: events ^b^	1%	2%	6%	8%	5%	7%	6%	9%
IDI: non-events ^c^	2%	4%	11%	19%	9%	15%	12%	20%
Overall IDI (95% CI), *p*-value	3% (1%, 6%), 0.02		17% (11%, 22%), <0.001		14% (8%, 18%), <0.001		18% (12%, 24%), <0.001	
Relative IDI	0.29		1.41		1.14		1.51	
Compared with VOE model								
IDI: events ^b^					3%	5%	5%	7%
IDI: non-events ^c^					7%	12%	9%	17%
Overall IDI (95% CI), *p*-value					10% (5%, 14%), <0.001		14% (9%, 20%), <0.001	
Relative IDI					0.65		0.94	

^a^ Variables adjusted in the reference model includes age, sex, and betel nut chewing; variables adjusted in VOE model include age, sex, betel nut chewing, and visual, oral examination results; variables adjusted in MET model include age, sex, betel nut chewing, and methylation marker; variables adjusted in triage model include age, sex, betel nut chewing, and an indicator to identify patients with positive results based on both visual and oral examination and methylation positivity; variables adjusted in co-testing model include age, sex, betel nut chewing, visual, oral examination results, and methylation marker; ^b^ events refer to biopsy-confirmed lesions with dysplastic or OSCC; ^c^ non-events refer to biopsy-confirmed lesions without dysplasia or OSCC diagnosis; OED, oral epithelial dysplasia; OSCC, oral squamous cell carcinoma; IDI, integrated discrimination improvement.

## Data Availability

Not applicable.
